# Report of Case

**Published:** 1880-03

**Authors:** H. H. Hurd

**Affiliations:** West De Pere, Wisconsin


### ARTICLE VII.

*Report of Case.*

May 14th, 1879, called to see Mrs. H. in labor with her fifth child. Confinement was expected about April 15th, at which time slight pain was experienced. Labor commenced several hours before a medical attendant was summoned. Patient had had pain every night for a week. Pulse, 102; temperature, 99°. Pain infrequent and very weak and the patient very much exhausted.

On examination found a body two inches in diameter projecting an inch and a half from the vulva. The finger, passed behind and in front of the body, determined with difficulty that there was no passage beyond it, demonstrating that it was the cervix uteri. The projecting portion was purple, and the os scarcely visible. The finger, when passed, gave a sensation as though it were being forced through clots of blood, and could not reach the presenting part, which the previous attempt had determined to be the head. There was no appearance of cancerous or other disease.

On inquiry, found that the patient had first noticed something wrong during the first month of pregnancy, when she felt something come down between the labia while at stool, which she replaced herself. Since that time it always came down if she strained at stool. Had consulted a neighboring physician without relief.

Being unable to replace the womb, I scarified it and applied warm fomentations, and attempted by various means (without ergot) to increase the uterine contraction. Failing in this, I sent for assistance, and when the patient was thoroughly anæsthetized applied the forceps. The bleeding from the cervix had so far relaxed the organ as to make this only moderately difficult. The child was delivered safely and easily, with apparently no injury to the mother except an expected laceration of the cervix, about one inch in length. The part was then returned within the body after the removal of the secundines.

May 15.—Patient doing well. Ordered that the vagina should be syringed out twice daily with dilute carbolic acid.

May 16.—Patient suffering much from pelvic pain. Tenderness of hypogastric region. Pulse, 120; temperature, 102°. Ordered opiate and continued injections.

From this time to June 16th the patient's general condition improved, though a tumor of considerable magnitude formed in the left iliac fossa. She then became impatient and put herself under the care of an "Indian Woman" before my next visit on the 23d. The "woman" spent the day in brewing two vile mixtures to be given in teacupful doses. One seemed to be intended to act on the uterus (which she supposed the tumor to be). The other produced violent catharsis.

On the evening of the 24th, at the urgent solicitation of a neighbor, I visited the patient again. Pulse almost undistinguishable; skin cold; fæces passed involuntarily every half hour. Tumor on steady pressure breaking up into smaller ones, and apparently partly formed by gas and fluids held back by the pressure on the intestine of the swelling in the iliac region.

Injected tr. opium in starch water, and gave brandy and ammonia liberally. The patient gradually gained strength, but was confined to the bed until late in August. The tumor was lanced above Poupart's ligament on July 10th, and opened of itself below that ligament, later in the month. The treatment is not given in full because the poverty of the family made food and the promotion of its assimilation the most essential medication.

Dec. 9th.—Patient consulted me as to a hernia formed in the site of the opening below Pouparte's ligament, but is otherwise in excellent health. At the last examination the cervix uteri was natural in size, but slightly deformed by the cicatrix of the laceration mentioned above. Menstruation normal.

WEST DE PERE, Wisconsin. DR. H. H. HURD.

